# Predictive values of ANGPTL8 on risk of all-cause mortality in diabetic patients: results from the REACTION Study

**DOI:** 10.1186/s12933-020-01103-7

**Published:** 2020-08-03

**Authors:** Huajie Zou, Yongping Xu, Xi Chen, Ping Yin, Danpei Li, Wenjun Li, Junhui Xie, Shiying Shao, Liegang Liu, Xuefeng Yu

**Affiliations:** 1 Division of Endocrinology, Department of Internal Medicine, Tongji Hospital, Tongji Medical College, Huazhong University of Science and Technology, 1095 Jiefang Avenue, Wuhan, 430030 China; 2 Department of Epidemiology and Biostatistics, School of Public Health, Tongji Medical College, Huazhong University of Science and Technology, Wuhan, China; 3 Computer Center, Tongji Hospital, Tongji Medical College, Huazhong University of Science and Technology, Wuhan, China; 4 Hubei Key Laboratory of Food Nutrition and Safety, Department of Nutrition and Food Hygiene, Tongji Medical College, Huazhong University of Science and Technology, Wuhan, China; 5 Ministry of Education Key Lab of Environment and Health, School of Public Health, Tongji Medical College, Wuhan, China

**Keywords:** ANGPTL8, Diabetes, Mortality, CVD

## Abstract

**Background:**

Angiopoietin-like protein 8 (ANGPTL8), an important regulator of lipid metabolism, is increased in diabetes and is associated with insulin resistance. However, the role of ANGPTL8 in the outcomes of diabetic patients remains unclear. This study aimed to investigate circulating levels of ANGPTL8 in participants with and without diabetes and its potential associations with clinical outcomes in a 5 year cohort study.

**Methods:**

Propensity-matched cohorts of subjects with and without diabetes from the Risk Evaluation of Cancers in Chinese Diabetic Individuals: A longitudinal (REACTION) study were generated on the basis of age, sex and body mass index at baseline. The primary outcome was all-cause mortality. The secondary outcomes were a composite of new-onset major adverse cardiovascular events, hospitalization for heart failure, and renal dysfunction (eGFR < 60/min/1.73 m^2^).

**Results:**

We identified 769 matched pairs of diabetic patients and control subjects. Serum ANGPTL8 levels were elevated in patients with diabetes compared to control subjects (618.82 $\pm$ 318.08 vs 581.20 $\pm$ 299.54 pg/mL, p = 0.03). Binary logistic regression analysis showed that elevated ANGPTL8 levels were associated with greater risk ratios (RRs) of death (RR in quartile 4 vs. quartile 1, 3.54; 95% CI 1.32–9.50) and renal dysfunction (RR in quartile 4 vs. quartile 1, 12.43; 95% CI 1.48–104.81) only in diabetic patients. Multivariable-adjusted restricted cubic spline analyses revealed a significant, linear relationship between ANGPTL8 and all-cause mortality in diabetic patients (p for nonlinear trend = 0.99, p for linear trend = 0.01) but not in control subjects (p for nonlinear trend = 0.26, p for linear trend = 0.80). According to ROC curve analysis, the inclusion of ANGPTL8 in QFrailty score significantly improved its predictive performance for mortality in patients with diabetes.

**Conclusion:**

Serum ANGPTL8 levels were associated with an increased risk of all-cause mortality and could be used as a potential biomarker for the prediction of death in patients with diabetes.

## Background

Diabetes is the fastest increasing disease worldwide and is a substantial threat to human health [1]. Among adults in China, diabetes was associated with twofold increased mortality compared with adults without diabetes [2]. Patients with diabetes are at high risk for adverse outcomes from cardiovascular disease (CVD) [3], heart failure (HF) [4] and renal disease [5]. Diabetes and its macrovascular and microvascular complications account for more than 2 million deaths every year [6] and constitute the seventh most common cause of disability worldwide [7]. The role of dyslipidaemia in the progression of the macrovascular and microvascular complications of diabetes has long been known [8, 9].

Angiopoietin-like protein 8 (ANGPTL8), also known as betatrophin, TD26, “refeeding induced in fat and liver” (RIFL), lipasin, and PRO1185, is a protein primarily produced in the liver and adipose tissue and it plays an important role in triglyceride (TG) metabolism [10]. ANGPTL8, together with ANGPTL3 and ANGPTL4, could regulate TG metabolism by inhibiting the activity of lipoprotein lipase (LPL) and subsequently elevating serum TG since LPL is an enzyme for TG hydrolysis and plasma TG clearance [11–13]. These studies indicated that ANGPTL8 is involved in lipid metabolism and the pathogenesis of atherosclerosis. ANGPTL8 levels were increased in diabetes [14–16], dyslipidaemia [17, 18], CVD [19], renal function [20–22], obesity [23], hypertension [24], and nonalcoholic fatty liver [25]. Furthermore, studies have revealed that ANGPTL8 levels also increased with age [26, 27], suggesting its potential association with aging and with age-related metabolic diseases.

Frailty, a state characterized by reduced physiological reserve and loss of resistance to stressors caused by accumulated age-related deficits, is a major health concern associated with aging [3, 4]. The importance of frailty is highlighted by its consistent association with all-cause mortality and adverse outcomes, such as institutionalization, physical limitations, disability, recurrent hospitalizations, falls and fractures [28, 29]. The frailty index (FI) is a well-established predictor of all-cause mortality [30] and other adverse health outcomes in older adults [31]. The FI has been used in many health services to assess hospital-acquired complications, worsening health, and loss of independence [4–9], as well as conditions such as cognitive impairment [10], heart disease [11], osteoporosis [12], intellectual disability [13], and systemic sclerosis [14]. It is also used in epidemiological and clinical studies to grade the degree of risk of several adverse outcomes, including mortality [32]. Hippisley-Cox et al. developed the FI as the QFrailty score and included further key determinants of death [33]. In this work, we retrospectively investigated circulating levels of ANGPTL8 in participants with and without diabetes and its potential associations with death and cardiovascular and renal outcomes in a 5 year cohort study. Furthermore, we evaluated the predictive value of QFrailty score in conjunction with the ANGPTL8 in the prediction of death.

## Materials and methods

### Study design and population

The present study participants were recruited from Hubei Province, China, from 2011 to 2012 as part of the Risk Evaluation of Cancer in Chinese Diabetic Individuals: a longitudinal (REACTION) study, which was conducted among 259,657 adults aged 40 years old and older in 25 communities across mainland China during 2011–2012 (baseline) and which invited participants to attend follow-up visits during 2014–2016 [34–37]. At baseline, a comprehensive set of questionnaires, clinical measurements, oral glucose tolerance tests (OGTTs), and laboratory examinations were conducted in all of the participants following standardized protocols. The diagnosis of diabetes was based on the American Diabetes Association 2014 criteria [38]. Diabetes was defined as fasting plasma glucose (FPG) concentration of 7.0 mmol/L or more, 2 h plasma glucose concentration (2 h PG) of 11.1 mmol/L or more, glycated haemoglobin A1c (HbA1c) of 6.5% or more, or a self-reported previous diagnosis of diabetes by health-care professionals. The duration of pre-existing diabetes was counted from the date of diagnosis, and the duration of newly diagnosed diabetes was counted as 0. Propensity scores were used to match subjects in the diabetic and control group (in a 1:1 ratio) with callipers of 0.05 using prespecified clinical variables, including age, sex and body mass index (BMI). Participants were excluded from the analysis if they had missing data for measures of glucose tolerance status at baseline or follow-up visits. The Committee on Human Research at Tongji Hospital, Tongji Medical College, Huazhong University of Science and Technology, approved the study protocol, and all of the participants provided written informed consent. All of the methods were performed in accordance with the relevant guidelines and regulations.

### Study outcomes

The primary outcome was death from all causes. The secondary outcomes were a composite of new-onset major adverse cardiovascular events (MACE), hospitalization for HF and renal dysfunction. MACE were defined as cardiovascular death, myocardial infarction, or ischaemic stroke [39]. Renal dysfunction was defined as a glomerular filtration rate (eGFR) less than 60 mL per minute per 1.73 m^2^ of body-surface area and new end-stage renal disease. All of these outcomes were confirmed by death certificates and hospital records.

### Clinical and biochemical evaluation

Data were collected from local community clinics at baseline and follow-up visits. As previously described in the REACTION study [37], information on sociodemographic characteristics, lifestyle factors, medical history and family history was collected by trained staff using a standard questionnaire. All of the participants were asked to fast for at least 10 h to undergo the OGTT and blood sampling for the analysis of various biochemical parameters. $\beta$ cell function was assessed by homeostasis model assessment of $\beta$ cell function (HOMA-$\beta$) [40]. Insulin resistance was estimated by the index of homeostasis model assessment of insulin resistance (HOMA-IR) [40].

### Laboratory tests of ANGPTL8

Blood samples were collected after overnight fasting. Serum was obtained after centrifugation and was aliquoted and then stored at − 80 °C. Fasting serum ANGPTL8 levels were assessed using ELISA kits (Eiaab Science, Wuhan, China; Catalogue No. E11644h) with an intra-assay coefficient of variation (CV) of $\le$ 6.5% and an interassay CV of $\le$ 9.2% (provided by the manufacturer). The procedures were performed in accordance with the manufacturer’s instructions. All of the samples were analysed in duplicate.

## Statistical analysis

Baseline characteristics of the participants are presented as the means ± standard deviation (SDs) or medians (interquartile ranges, IQRs) for continuous variables and numbers (proportions) for categorical variables. The highest and lowest 0.5% for ANGPTL8 was trimmed [41]. We obtained p values using the Kruskal–Wallis test for continuous variables after passing Shapiro–Wilk normalization testing and the *χ*^2^ test for categorical variables. Correlations between variables were assessed using Pearson’s correlation analysis by controlling for the covariates, including age, sex, BMI and lipid profiles. Binary logistic regression analysis was conducted to calculate risk ratios (RRs) and 95% confidence intervals (CIs) for outcomes in quartiles of ANGPTL8. Potential nonlinear relationships between the levels of ANGPTL8 and the incidence of clinical outcomes were examined with restricted cubic splines [42]. A knot was located at the 25th, 50th, and 75th percentiles for ANGPTL8. Tests for nonlinearity were conducted using likelihood ratio tests. If a test for nonlinearity was not significant, we conducted a test for linearity. The QFrailty score (predictor variables are shown in Additional file 1. Appendix 1) was used as a standard criterion to quantify the absolute risk of death in the general population [33]. Receiver-operator characteristic (ROC) curves were drawn, and the performance of the model was evaluated by the area under the curve (AUC). Comparison of p values was performed using Medcalc ROC analysis software. A 2-tailed p value < 0.05 was considered significant. SPSS software (version 20.0), and Stata software (version 12.0) were used for all of the analyses.

## Results

### Baseline characteristics

Among 10,999 study participants, 9221 (83.8%) were followed up during 2011–2016. Of these participants, 1058 patients were diagnosed with diabetes. We excluded 277 patients with one or more glycaemic measures missing at baseline. Additionally, the highest and lowest 0.5% for ANGPTL8 was trimmed, leaving 781 patients for analysis. After the propensity matching, we acquired 769 matched pairs of diabetic patients and control subjects (Additional file 1. Figure S1). In diabetic patients, 60.6% (N = 466) were newly diagnosed with diabetes, and 39.4% (N = 303) had pre-existing diabetes (Additional file 1. Table S1). Only 19.5% (N = 150) of patients were treated with oral antidiabetic drugs (OADs) or insulin. In patients with medications, 86.6% patients were treated with OADs, 4.7% patients with insulin and 10.7% with a combination of OADs and insulin.

We found that serum ANGPTL8 levels were elevated in diabetic patients compared to control subjects (618.82 $\pm$ 318.08 vs 581.20 $\pm$ 299.54 pg/mL, p = 0.03, Additional file 1. Table S2). Furthermore, levels of BMI, HbA1c, FPG, 2 h PG, fasting insulin, HOMA-IR, low density lipoprotein (LDL), TG, cholesterol, creatinine, and incident rates of hypertension and hyperlipidaemia in diabetic patients were higher than in the control subjects (all p < 0.05, Additional file 1. Table S2). During up to 5 years of follow-up, there were 19 participants (2.5%) who died and 44 (5.7%) incident cases for the secondary outcomes among 769 control subjects. The incidence of death (N = 56, 7.3%) and the secondary outcomes (N = 91, 11.8%) were increased in patients with diabetes (all p < 0.05, Additional file 1. Table S2).

Baseline characteristics of participants with and without diabetes according to quartiles of ANGPTL8 are presented in Table 1. Age, sex, aspartate aminotransferase (AST), creatinine, eGFR and smoking status changed with ANGPTL8 levels in both the diabetic patients and control subjects (all p values < 0.05). In control subjects, ANGPTL8 levels were also associated with waist-hip ratio (WHR), systolic blood pressure (SBP), HbA1c and incidence of hypertension (all p values < 0.05). ANGPTL8 levels were associated with BMI high-density lipoprotein (HDL) and incidence of hyperlipidaemia in the diabetic patients (all p values < 0.05).

**Table 1:** Clinical and biochemical parameters for control subjects and diabetic patients, according to quartile of ANGPTL8 levels

Characteristics	Control (ANGPTL8, pg/mL)				*p* value	Diabetes (ANGPTL8, pg/mL)				*p* value
	Q1 (< 372.15)	Q2 (372.15–537.58)	Q3 (537.59–74.09)	Q4 (> 746.09)		Q1 (< 347.15)	Q2 (347.15–534.23)	Q3 (534.24–810.50)	Q4 (> 810.50)	
N	192	193	192	192		192	193	192	192	
ANGPTL8 (pg/ml)	261.28 (188.24–317.25)	432.03 (403.26–476.68)	623.40 (573.62–690.53)	959.43 (850.77–1143.01)	< 0.001	256.45 (189.45–300.93)	462.97 (406.66–496.93)	642.52 (586.97–729.70)	1009.54 (893.07–1268.33)	< 0.001
Age (years)	54.44 $\pm$ 9.03	56.97 $\pm$ 8.75	59.54 $\pm$ 8.91	64.87 $\pm$ 9.82	< 0.001	59.14 $\pm$ 9.56	58.30 $\pm$ 9.37	61.33 $\pm$ 9.21	63.57 $\pm$ 9.16	< 0.001
Male (%)	47 (24.5)	59 (30.6)	68 (35.4)	92 (47.9)	< 0.001	40 (20.8)	59 (30.6)	68 (35.4)	99 (51.6)	< 0.001
BMI (kg/m^2^)	23.39 $\pm$ 3.16	23.05 $\pm$ 3.03	23.35 $\pm$ 2.93	23.71 $\pm$ 3.21	0.24	24.37 $\pm$ 3.50	24.79 $\pm$ 3.44	24.10 $\pm$ 3.23	23.62 $\pm$ 3.67	0.009
WHR	0.84 (0.80–0.88)	0.85 (0.81–0.89)	0.85 (0.81–0.90)	0.87 (0.83–0.90)	0.005	0.88 (0.85–0.91)	0.88 (0.84–0.93)	0.88 (0.84–0.92)	0.88 (0.84–0.91)	0.53
SBP	139 (127–156)	145 (129–162)	146 (130–164)	151 (135–168)	< 0.001	150 (136–168)	149 (135–166)	155 (138–173)	154 (137–173)	0.33
HbA1c (%)	5.50 (5.30–5.80)	5.50 (5.30–5.70)	5.60 (5.40–5.80)	5.60 (5.40–5.90)	0.003	6.50 (5.80–7.70)	6.50 (5.85–8.15)	6.55 (5.90–8.15)	6.40 (5.73–7.80)	0.62
FPG (mmol/L)	5.04 (4.75–5.34)	5.02 (4.77–5.28)	5.00 (4.72–5.26)	5.06 (4.80–5.31)	0.39	7.18 (5.95–8.13)	7.26 (6.19–9.02)	7.37 (6.05–8.98)	7.35 (5.98–8.66)	0.38
2 h PG (mmol/L)	5.72 (4.92–6.42)	5.83 (5.01–6.56)	5.72 (4.91–6.44)	5.84 (5.20–6.63)	0.18	11.38 (7.93–13.72)	12.17 (7.77–15.70)	12.12 (8.31–14.38)	12.06 (9.24–15.74)	0.09
Fasting insulin (pmol/mL)	5.00 (3.50–7.08)	4.80 (3.20–6.90)	4.80 (3.40–7.10)	4.80 (3.40–7.00)	0.58	6.80 (4.30–12.50)	7.10 (4.25–10.95)	6.85 (4.40–10.45)	6.15 (3.63–11.18)	0.48
HOMA-IR	1.19 (0.77–1.62)	1.09 (0.70–1.52)	1.06 (0.74–1.59)	1.06 (0.77–1.59)	0.72	2.24 (1.41–4.04)	2.39 (1.33–4.13)	2.42 (1.34–3.98)	2.17 (1.15–4.00)	0.59
HOMA-$\beta$(%)	66.221 (45.56–89.32)	61.76 (44.59–91.01)	70.91 (44.32–100.00)	59.98 (42.72–84.54)	0.20	41.76 (23.60–73.88)	38.76 (18.13–73.84)	36.27 (22.39–63.55)	36.14(17.70–65.55)	0.31
Fasting HDL (mmol/L)	1.37 (1.14–1.60)	1.38 (1.17–1.60)	1.36 (1.15–1.61)	1.34 (1.13–1.61)	0.94	1.44 (1.24–1.70)	1.35 (1.17–1.61)	1.35 (1.16–1.58)	1.32 (1.11–1.62)	0.03
Fasting LDL (mmol/L)	2.43 (2.06–2.96)	2.58 (2.11–3.06)	2.59 (2.10–3.06)	2.62 (2.17–3.04)	0.25	2.93 (2.37–3.64)	2.97 (2.36–3.54)	3.03 (2.42–3.61)	2.83 (2.25–3.50)	0.20
Fasting TG (mmol/L)	1.19 (0.87–1.63)	1.18 (0.90–1.73)	1.16 (0.86–1.74)	1.23 (0.89–1.74)	0.45	1.46 (1.04–2.17)	1.46 (0.95–2.10)	1.49 (1.07–2.08)	1.49 (1.00–2.55)	0.67
Cholesterol (mmol/L)	4.58 (3.92–5.24)	4.67 (4.15–5.30)	4.61 (4.08–5.37)	4.86 (4.19–5.44)	0.10	5.16 (4.57–6.16)	5.13 (4.47–5.85)	5.14 (4.48–5.90)	5.10 (4.38–5.98)	0.78
ALT (U/L)	13 (10–18)	13 (10–17)	14 (11–18)	14 (10–18)	0.48	12 (9–18)	13 (10–19)	14 (10–21)	14 (10–22)	0.05
AST (U/L)	21 (17–25)	23 (19–26)	23 (20–27)	23 (20–28)	< 0.001	20 (17–26)	21 (17–26)	21 (17–28)	23 (18–31)	0.007
eGFR (mL/min/1.73 m^2^)	119.54 (107.40–134.39)	119.41 (105.77–130.13)	116.88 (101.22–128.33)	108.12 (92.58–122.90)	< 0.001	113.51 (100.39–120.63)	111.67 (100.23–120.27)	108.58 (97.16–119.44)	106.71 (90.35–118.79)	0.008
Creatinine (μmol/L)	58.10 (52.33–64.85)	58.50 (53.40–64.45)	61.05 (55.43–66.83)	65.45 (57.18–72.98)	< 0.001	60.80 (55.90–65.48)	62.00 (56.90–68.05)	63.10 (58.13–69.05)	66.60 (59.85–74.68)	< 0.001
Currently smoking	19 (10.2)	36 (18.8)	20 (10.6)	43 (23.0)	0.001	15 (8.7)	20 (10.9)	29 (16.1)	50 (26.5)	< 0.001
Hypertension	96 (50.0)	119 (61.7)	122 (63.5)	141 (73.4)	< 0.001	143 (74.5)	142 (73.6)	146 (76)	152 (79.2)	0.59
Hyperlipidaemia	62 (32.2)	69 (35.8)	72 (37.0)	85 (44.3)	0.10	113 (58.9)	105 (54.4)	93 (48.4)	124 (64.6)	0.01

*BMI* body-mass index, *WHR* waist hip rate, *SBP* systolic blood pressure, *HbA1c* glycated haemoglobin A1c, *FPG* fasting plasma glucose, *2 h PG* 2 h plasma glucose concentration, *HOMA-IR* homeostasis model assessment of insulin resistance, *HOMA*-β, homeostasis model assessment of β cell function, *HDL* high density lipoprotein, *LDL* low density lipoprotein, *TG* triglycerides, *ALT* alanine transaminase, *AST* aspartate aminotransferase, *Egfr* glomerular filtration rate

Next, we studied correlations between ANGPTL8 levels and related variables in control subjects and diabetic patients using Pearson’s correlation analysis. After controlling for multiple variables, ANGPTL8 levels positively correlated with age (r = 0.42), BMI (r = 0.10), and creatinine (r = 0.11) but inversely correlated with eGFR (r = −0.12) in the control subjects (all p values < 0.05) (Additional file 1. Table S3, model 3). Moreover, ANGPTL8 levels positively correlated with age (r = 0.18), duration of diabetes (r = 0.08), 2 h PG (r = 0.08), alanine transaminase (ALT) (r = 0.07), AST (r = 0.13) and creatinine (r = 0.10) but inversely correlated with BMI (r = − 0.07), HDL (r = − 0.09) and eGFR (r = − 0.13) in diabetic patients (all p values < 0.05) (Additional file 1. Table S3, model 3). The positive correlation of ANGPTL8 with TG (p = 0.001, model 2) was also observed, although it diminished after adjusting for other lipid profiles (p = 0.41, model 3).

### ANGPTL8 correlates with all-cause mortality and renal dysfunction

As shown in Table 2, increasing quartiles of ANGPTL8 were associated with elevated incidences of death and renal dysfunction in the diabetic patients (all p values < 0.05) but not in the control subjects. Furthermore, death due to CVD in diabetic patients also increased numerically in the highest quartile of ANGPTL8 levels (p = 0.06, Table 2). Binary logistic regression analyses showed that, compared with the first quartile, non-adjusted RRs (96% CIs) (model 1, Table 3) for the primary outcome (all-cause mortality) were 4.67 (1.00–21.92) and 4.64 (1.86–11.59) for the fourth ANGPTL8 quartile in the control subjects and diabetic patients, respectively. The non-adjusted RRs (96% CIs) (model 1, Table 3) for the secondary outcomes were 2.57 (1.04–6.34) and 1.72 (0.95–3.12), respectively, for the fourth ANGPTL8 quartile compared with the first quartile in the control subjects and diabetic patients. However, the associations of ANGPTL8 with all-cause mortality only persisted in the diabetic patients, although they were slightly attenuated after additional adjustment for covariables, including age, sex and BMI (RR, 3.59; 95% CI 1.36–9.51; model 2) and further adjustment for lipid profiles and duration and treatment of diabetes (RR, 3.54; 95% CI 1.32–9.50; model 3). Then, we further analysed the association of ANGPTL8 with any single component of the secondary outcomes and found that elevated ANGPTL8 was associated with an increased risk for renal dysfunction in the diabetic patients (RR in quartile 4 vs. quartile 1, 12.43; 95% CI 1.48–104.81; Table 3, model 3) after adjusting for covariables.

**Table 2:** Outcomes in control subjects and diabetic patients, according to quartile of ANGPTL8 level

Outcomes— no. (%)	Control				*p* value	Diabetes				*p* value
	Q1	Q2	Q3	Q4		Q1	Q2	Q3	Q4	
Primary outcome										
Death	2 (1.0)	3 (1.6)	5 (2.6)	9 (4.7)	0.10	6 (3.1)	12 (6.2)	13 (6.8)	25 (13.0)	0.002
Secondary outcomes										
	7 (3.6)	10 (5.2)	10 (5.2)	17 (8.9)	0.16	20 (10.4)	21 (10.9)	18 (9.4)	32 (16.7)	0.11
CVD death	1 (0.5)	1 (0.5)	2 (1.0)	6 (3.1)	0.08	3 (1.6)	9 (4.7)	6 (3.1)	13 (6.8)	0.06
MI	1 (0.5)	2 (1.0)	0 (0)	7 (3.6)	0.01	3 (1.6)	6 (3.1)	4 (2.1)	11 (5.7)	0.09
Stroke	3 (1.6)	7 (3.6)	7 (3.6)	6 (3.1)	0.59	12 (6.3)	8 (4.1)	6 (3.1)	8 (4.1)	0.50
HF	1 (0.5)	1 (0.5)	0 (0)	1 (0.5)	0.80	4 (2.1)	2 (1.0)	2 (1.0)	4 (2.1)	0.71
Renal dysfunction	2 (1.0)	0 (0)	2 (1.0)	3 (1.6)	0.43	1 (0.5)	4 (2.1)	5 (2.6)	13 (6.8)	0.003

*CVD* cardiovascular disease, *MI* myocardial infarction, *HF* hospitalization for heart failure

**Table 3:** Risk ratios for clinical outcomes according to quartiles of ANGPTL8

Outcome		Control			Diabetes		
		Model 1	Model 2	Model 3	Model 1	Model 2	Model 3
Primary outcome							
Death	Q1 (Reference)	1	1	1	1	1	1
	Q2 (RR, 95% CI)	1.50 (0.25–9.08)	1.32 (0.21–8.52)	1.26 (0.20–8.03)	2.06 (0.76–5.59)	2.61 (0.90–7.59)	2.43 (0.83–7.16)
	Q3 (RR, 95% CI)	2.54 (0.49–13.23)	1.56 (0.28–8.70)	1.48 (0.27–8.23)	2.25 (0.84–6.05)	2.06 (0.72–5.86)	2.08 (0.72–6.02)
	Q4 (RR, 95% CI)	4.67 (1.00–21.92)	1.47 (0.28–7.64)	1.32 (0.25–7.06)	4.64 (1.86–11.59)	3.59 (1.36–9.51)	3.54 (1.32–9.50)
Secondary outcomes							
	Q1 (Reference)	1	1	1	1	1	1
	Q2 (RR, 95% CI)	1.44 (0.54–3.88)	1.26 (0.46–3.42)	1.21 (0.44–3.30)	1.05 (0.55–2.01)	1.07 (0.55–2.09)	1.05 (0.53–2.06)
	Q3 (RR, 95% CI)	1.45 (0.54–3.90)	1.05 (0.38–2.87)	1.02 (0.37–2.80)	0.89 (0.46–1.74)	0.75 (0.38–1.51)	0.72 (0.35–1.45)
	Q4 (RR, 95% CI)	2.57 (1.04–6.34)	1.28 (0.48–3.40)	1.19 (0.45–3.16)	1.72 (0.95–3.12)	1.30 (0.69–2.44)	1.20 (0.63–2.28)
MACE	Q1 (Reference)	1	1	1	1	1	1
	Q2 (RR, 95% CI)	2.30 (0.70–7.60)	2.02 (0.61–6.73)	1.96 (0.58–6.56)	0.99 (0.47–2.10)	1.05 (0.48–2.28)	1.01 (0.46–2.21)
	Q3 (RR, 95% CI)	1.78 (0.51–6.18)	1.33 (0.38–4.70)	1.29 (0.36–4.57)	0.79 (0.36–1.73)	0.65 (0.29–1.48)	0.65 (0.29–1.49)
	Q4 (RR, 95% CI)	3.70 (1.19–11.44)	2.02 (0.61–6.72)	1.90 (0.57–6.27)	1.37 (0.68–2.77)	0.99 (0.48–2.08)	0.98 (0.46–2.09)
HF	Q1 (Reference)	1	1	1	1	1	1
	Q2 (RR, 95% CI)	1.00 (0.06–16.02)	1.62 (0.07–38.32)	0.93 (0.04–23.90)	1.20 (0.36–4.00)	1.10 (0.32–3.78)	1.11 (0.32–3.83)
	Q3 (RR, 95% CI)	–	–	–	0.80 (0.21–3.01)	0.62 (0.16–2.40)	0.60 (0.15–2.40)
	Q4 (RR, 95% CI)	1.00 (0.06–16.10)	0.23 (0.01–5.84)	0.14 (0.01–4.21)	2.06 (0.69–6.13)	1.39 (0.45–4.30)	1.36 (0.43–4.32)
Renal dysfunction	Q1 (Reference)	1	1	1	1	1	1
	Q2 (RR, 95% CI)	–	–	–	4.04 (0.45–36.50)	4.08 (0.45–37.01)	4.43 (0.47–41.99)
	Q3 (RR, 95% CI)	1.00 (0.14–7.17)	0.90 (0.12–6.75)	0.92 (0.12–7.15)	5.11 (0.59–44.13)	4.85 (0.56–42.13)	4.89 (0.53–44.95)
	Q4 (RR, 95% CI)	1.51 (0.25–9.13)	1.22 (0.16–9.26)	1.05 (0.13–8.54)	13.87 (1.80–107.12)	12.53 (1.59–98.90)	12.43 (1.48–104.81)

Model 1 was unadjusted

Model 2 was adjusted for age, sex and BMI

Model 3 was adjusted for all variables in model 2 plus HDL, LDL, TG, cholesterol, duration of diabetes and treatment of diabetes in diabetes group

*MACE* new-onset major adverse cardiovascular events, *HF* hospitalization for heart failure

**Fig. 1:**
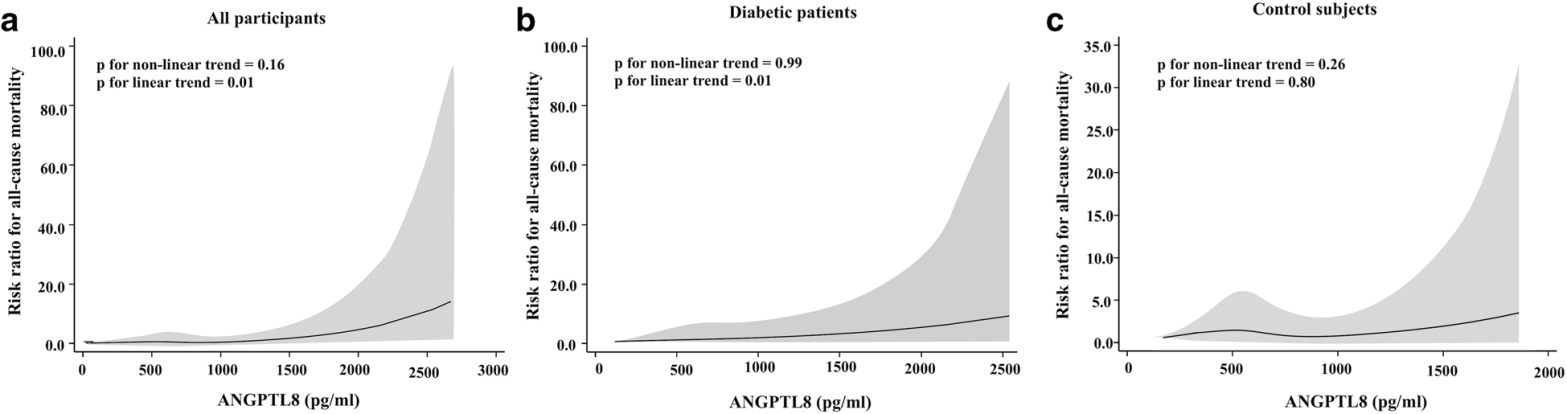
Multivariable-adjusted risk ratios for all-cause mortality in all participants (**a**), diabetic patients (**b**) and controls (**c**). The solid lines indicate multivariate-adjusted risk ratios, and the shaded area indicate the 95% CIs derived from restricted cubic spline regression. A knot is located at the 25, 50, and 75th percentiles for ANGPTL8, and the highest and lowest 0.5% of ANGPTL8 was trimmed. The logistic regression was adjusted for age, sex, and BMI

Multivariable-adjusted restricted cubic spline analyses suggested a linear relationship of ANGPTL8 with all-cause mortality in all of the participants (p for nonlinear trend = 0.16, p for linear trend = 0.01; Fig. 1a). Further analysis indicated a significant linear relationship between ANGPTL8 and all-cause mortality in diabetic patients (p for nonlinear trend = 0.99, p for linear trend = 0.01; Fig. 1b) but not in control subjects (p for nonlinear trend = 0.26, p for linear trend = 0.80; Fig. 1c) after adjusting for age, sex, BMI and lipid profiles.

### Predictive values of ANGPTL8 for death

**Fig. 2:**
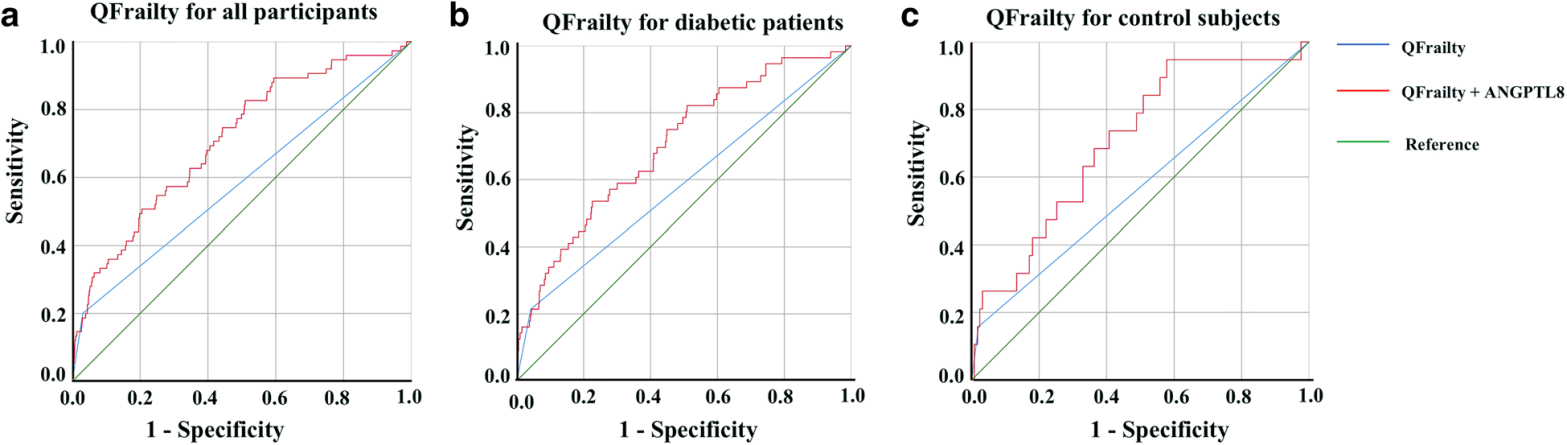
Comparison of predictive performances for all-cause mortality between a combination of serum ANGPTL8 levels with QFrailty score and QFrailty score alone in all of the participants (**a**), diabetic patients (**b**) and controls (**c**)

We observed that, when ANGPTL8 levels were combined with QFrailty score, there was improvement for death prediction compared with the QFrailty score alone (Fig. 2a–c, Additional file 1. Table S4). The AUC for the ANGPTL8 + QFrailty model was 0.71 vs 0.59 for the QFrailty score alone (*p* < 0.001; Fig. 2a) in all of the participants. Consistently, the inclusion of ANGPTL8 improved the predictive performance of QFrailty score in diabetic patients (AUC 0.70 vs 0.59; *p* < 0.001; Fig. 2b) and control subjects (AUC 0.71 vs 0.57; *p* = 0.01; Fig. 2c).

## Discussion

This retrospective cohort study found that ANGPTL8 levels were elevated and independently associated with all-cause mortality and renal dysfunction in patients with diabetes. The linear relationship of ANGPTL8 with mortality was only observed in the patients with diabetes. The elevated levels of ANGPTL8 were correlated with risk factors related to mortality, such as sex, age, BMI, smoking, eGFR, creatinine, and hyperlipidaemia. Furthermore, the inclusion of ANGPTL8 in QFrailty score significantly improved its predictive performance for mortality in patients with diabetes.

### ANGPTL8 levels are increased in patients with diabetes and accompanied by elevated levels of factors associated with metabolic disorders

In our study, diabetic patients had higher ANGPTL8 levels, accompanied by increased incident rates of metabolic disorders, such as hyperlipidaemia, hyperglycaemia and hypertension, consistent with findings from previous observational studies [14–16]. Increased insulin levels in patients with diabetes, as shown in our study, are among the important factors for stimulating ANGPTL8 production [16]. The results from the present study that ANGPTL8 was positively correlated with TG and negatively correlated with HDL in diabetic patients implied that ANGPTL8 could make a partial contribution to dyslipidaemia by affecting lipid metabolism. This effect, combined with abnormal metabolism of glucose, high incident rates of hypertension and renal dysfunction, and increased BMI, might contribute together to atherosclerosis, CVD and death in diabetic patients. ANGPTL8 is related to HDL-C dysfunction and is involved in the association between dyslipidaemia and arteriosclerosis [27], regardless of glucose intolerance or diabetes mellitus [43, 44]. ANGPTL8 presents a negative effect on HDL-mediated cholesterol efflux capacity [17] and a strong link with subclinical atherosclerosis [27], and its levels are significantly increased in patients with coronary disease, proportional to disease severity [45]. However, underlying mechanisms for these pathophysiology associations await expounding. Regarding genetics and molecular pathways, miR-143-3p regulates expression of ANGPTL8 transcript and protein levels [46], the prevalence of T2DM and impaired glucose tolerance is greater in subjects with the R59W ANGPTL8 variant [47], and the concomitant presence of CETP B1, NOS3 T and ANGPTL8 T alleles augments the risk of CVD and T2DM [48]. Furthermore, it was reported that targeting ANGPTL8 with antisense oligonucleotide (ASO), a second-generation 2-O-methoxyethyl ASO against ANGPTL8, could prevent cardiovascular disorders [49]. Moreover, GLP-1R agonists stimulate ANGPTL8 production in human hepatocytes dose-dependently [50], and ANGPTL8 has been described as a novel vitamin D receptor target gene involved in nonalcoholic fatty liver pathogenesis [25]. In contradiction with the mainstream conceptions, it has been recently argued that a high ANGPTL8 level in coronary patients protects them from cardiovascular events [51]. ANGPTL8, therefore, has extensive involvement in clinical outcomes and plays critical roles in the progression of complications of cardiometabolic diseases.

### All-cause mortality is increased with ANGTPL8 levels and the inclusion of ANGPTL8 in QFrailty score improves its performance for death prediction

We found that the incidence of death increased in participants with diabetes compared to control subjects, consistent with previous publications [2, 52]. Furthermore, a linear relationship between ANGPTL8 levels and risk for all-cause mortality in diabetic patients, but not in control subjects, clearly indicated that ANGPTL8 was a strong predictor of all-cause mortality in patients with diabetes. Further analysis revealed that inclusion of ANGPTL8 in QFrailty score improved the predictive performance for death compared with the QFrailty score alone in diabetic patients. Diabetes is associated with increased risk of death from a wide range of causes, including diabetes-related increased prevalence of microvascular and macrovascular diseases, cancers, infections, liver disease, obesity, and external causes [52]. Our study suggests that elevated levels of ANGPLT8 might actually be one of important risk factors for diabetic patients and might be used in combination with other factors to detect high-risk patients. In diabetic patients, hyperglycaemia and altered lipid profiles are associated with diabetic nephropathy development [20, 53, 54]. The present study showed that ANGPTL8 was inversely correlated with eGFR and independently associated with renal dysfunction in diabetic patients, consistent with previous publications [20], and might also contribute to all-cause mortality [55].

It is interesting to note that inclusion of ANGPTL8 in QFrailty score also improved its predictive performance for death compared with the QFrailty score alone in control subjects. However, no detection of an association of ANGPTL8 with death or a linear relationship between ANGPTL8 levels and risk of all-cause mortality in the control subjects might suggest that further studies are needed to explore the role of ANGPTL8 in death prediction in the general population.

## Limitations

There are some limitations of this study. First, all of the participants in our study were Chinese, limiting the generalizability of the findings, which must be confirmed in other ethnic groups. Second, although we used propensity scores to match subjects in the diabetic and control subjects, the retrospective design might also have carried the risk of selection bias; therefore, prospective studies are needed to confirm the results. Regardless, this study is the first report showing that ANGPTL8 could be a potential biomarker for death prediction in patients with diabetes. Third, our study could not identify the causal mechanisms driving the observed associations between ANGPTL8 and detrimental outcomes. Future physiological studies are needed for the causal links and underlying mechanisms. Finally, the study participants were followed for only 5 years. This relatively short follow-up duration reduced the number of clinical events and the study’s statistical power, especially for determining CVD mortality and CVD events. Future studies should be conducted in larger sample sizes to verify the findings of our study.

## Conclusion

In conclusion, serum ANGPTL8 levels were associated with an increased risk of all-cause mortality and renal dysfunction in patients with diabetes. Furthermore, inclusion of ANGPTL8 improved the performance of QFrailty scores in predicting all-cause mortality; therefore, serum ANGPTL8 levels might be used as an important biomarker for the prediction of death in patients with diabetes. These findings might suggest that ANGPTL8 is a novel regulator for metabolic diseases with adverse outcomes. Future studies are needed to evaluate ANGPTL8 as a biomarker for the risk of death in cardiometabolic diseases, including diabetes and to study the underlying mechanisms for its potential role in adverse health outcomes in these diseases.

## Supplementary information

**Additional file 1: Appendix 1.** Predictor variables for QFrailty score. **Table S1.** Characteristics of patients with diabetes. **Table S2.** Metabolic parameters and outcomes for control subjects and diabetic patients. **Table S3.** Partial correlations between ANGPTL8 levels and clinical variables in control subjects and diabetic patients. **Table S4.** Predictive values for all-cause mortality in combination with ANGPTL8 in QFrailty score. **Figure S1.** Flow diagram for the study population selection.

## Data Availability

The datasets used and/or analysed during the current study are available from the corresponding author on reasonable request.

## Abbreviations

ALT: Alanine transaminase
AST: Aspartate aminotransferase
AUC: Areas under the curve
BMI: Body-mass index
CI: Confidence intervals
eGFR: Glomerular filtration rate
FPG: Fasting plasma glucose
2 h PG: 2 h plasma glucose concentration
HbA1c: Glycated haemoglobin A1c
HDL: High density lipoprotein
HOMA-IR: Homeostasis model assessment of insulin resistance
HOMA-*β*: Homeostasis model assessment of β cell function
LDL: Low density lipoprotein
MACE: Major adverse cardiovascular events
OAD: Oral antidiabetic drug
ROC: Receiver-operator characteristic
RR: Risk ratio
SBP: Systolic blood pressure
TG: Triglycerides
WHR: Waist hip rate
