# Supplementary material for: Predictive values of ANGPTL8 on risk of all-cause mortality in diabetic patients: results from the REACTION Study
Source: Cardiovasc Diabetol. 2020 Aug 3;19:121. doi: 10.1186/s12933-020-01103-7 (PMC7398345; doi:10.1186/s12933-020-01103-7)

**Supplementary Online Content**

Huajie Zou, Yongping Xu, et al. Predictive Values of ANGPTL8 on Risk of All-cause Mortality in Diabetic patients: results from the REACTION Study

Appendix 1. Predictor variables for QFrailty score.

Table S1. Characteristics of patients with diabetes.

Table S2. Metabolic parameters and outcomes for control subjects and diabetic patients.

Table S3. Partial correlations between ANGPTL8 levels and clinical variables in control subjects and diabetic patients.

Table S4. Predictive values for all-cause mortality in combination with ANGPTL8 in QFrailty score.

Figure S1. Flow diagram for the study population selection.

***Correspondence**:

Xuefeng Yu, M.D., Ph.D., Professor of Medicine, Division of Endocrinology, Department of Internal Medicine, Tongji Hospital, Tongji Medical College, Huazhong University of Science and Technology, 1095 Jiefang Avenue, Wuhan 430030, China

Email: xfyu188@163.com

Tel./Fax: +86 027 83662883.

Appendix 1: Predictor variables for QFrailty score

• Age (continuous variable)

• Geographical region in the United Kingdom (10 regions)

• Townsend deprivation score. This score is an area-level continuous score based on the patients’ postcode.11 Originally developed by Townsend,11 the score includes unemployment (as a percentage of those aged 16 years old or older who are economically active), non-car ownership (as a percentage of all households), non-home ownership (as a percentage of all households), and household overcrowding. These variables are measured for a given area of approximately 120 households using the 2011 census, and they are combined to yield a Townsend score for the area. A higher Townsend score implies a greater level of deprivation

• Ethnic group (nine categories)

• Alcohol intake (<1 unit/day, 1-2 units/day, 3-6 units/ day, 7-9 units/day, ≥9 units/day)

• Smoking status (non-smoker; former smoker; light, moderate, or heavy smoker)

• Body mass index (continuous variable)

• Unplanned admissions in past 12 months (0, 1, 2, or ≥3) as recorded in the linked hospital data

• Poor mobility (poor mobility, housebound, confined to chair, bedridden, requires home visits, receives mobility allowance)

• Lives in a care home (nursing home or residential care)

• Lives alone

• Atrial fibrillation

• Congestive heart failure

• Cardiovascular disease (myocardial infarction, angina, stroke, or transient ischaemic attack)

• Valvular heart disease

• Peripheral vascular disease

• Treated hypertension (hypertension and current antihypertensive treatment)

• Chronic kidney disease (stages 4 or 5)

• Diabetes (none, type 1, type 2)

• Hypothyroidism

• Hyperthyroidism

• Cancer

• Chronic liver disease or pancreatitis

• Malabsorption (including Crohn’s disease, ulcerative colitis, coeliac disease, steatorrhoea, blind loop syndrome)

• Peptic ulcer (gastric or duodenal ulcer, simple or complicated ulcer)

• Asthma or chronic obstructive airways disease

• Epilepsy

• Dementia

• Learning disability

• Osteoporosis

• Fragility fracture (hip, spine, shoulder, or wrist fracture)

• Parkinson’s disease or syndrome

• Rheumatoid arthritis

• Falls

• Bipolar disorder or schizophrenia

• Depression in past 12 months

• Venous thromboembolism

• Anaemia (haemoglobin <110 g/L)

• Abnormal liver function test result (bilirubin, alanine aminotransferase, or γ glutamyl transferase more than three times the upper limit of normal)

• High platelet count (>480×109/L)

• Leg ulcer (leg, shin, ankle or foot ulcer, ischaemic neuropathic, arterial, or venous ulcer)

• Blindness (registered blind or partially sighted or visual impairment)

• Appetite loss in past 12 months

• Weight loss in past 12 months (unexplained or abnormal weight loss)

• Urinary incontinence in past 12 months

• Nocturia in past 12 months

• Urinary retention in past 12 months (acute or chronic retention)

• Syncope (vasovagal symptom, faint, collapse, “funny turn,” drop attack) in past 12 months

• Dizziness in past 12 months

• Insomnia in past 12 months

• Dyspnoea in past 12 months (breathless at rest or on exertion, paroxysmal nocturnal dyspnoea)

• Hearing impairment or deafness in past 12 months

• Loneliness in past 12 months

• Use of anticoagulants (≥2 prescriptions in past six months)

• Use of antidepressants (≥2 prescriptions in past six months)

• Use of antipsychotics (≥2 prescriptions in past six months)

• Use of corticosteroids (≥2 prescriptions in past six months)

• Non-steroidal anti-inflammatory drugs (≥2 prescriptions in past six months)

**Table S1.** **Characteristics of patients with diabetes.**

|  | Pre-existing diabetes | | | | Newly diagnosed diabetes | p^2^ value |
| --- | --- | --- | --- | --- | --- | --- |
|  | With treatment | Without treatment | p^1^ value | All |  |  |
| N (%) | 150 (19.5) | 153 (19.9) | - | 303 (39.4) | 466 (60.6) | - |
| ANGPTL8 (pg/mL) | 648.38$\pm$27.75 | 592.01$\pm$30.20 | 0.09 | 619.92$\pm$20.56 | 618.11$\pm$18.34 | 0.42 |
| **Primary outcome** | | | | | | |
| Death | 8 (5.3) | 9 (5.9) | 1.00 | 17 (5.6) | 39 (8.4) | 0.16 |
| **Secondary outcomes** | 15 (10.0) | 22 (14.4) | 0.29 | 37 (12.2) | 54 (11.6) | 0.82 |
| MACE | 10 (6.7) | 15 (9.8) | 0.41 | 25 (8.3) | 37 (7.9) | 0.89 |
| HF | 3 (2.0) | 7 (4.6) | 0.34 | 10 (3.3) | 15 (3.2) | 0.95 |
| Renal dysfunction | 4 (2.7) | 4 (2.6) | 1.00 | 8 (2.6) | 15 (3.2) | 0.83 |

Abbreviation: MACE, new-onset major adverse cardiovascular events; HF, hospitalization of heart failure.

p^1^for significance of comparison between diabetic patients with or without treatment.
p^2^for significance of comparison between patients with newly diagnosed diabetes and patients with pre-existing diabetes.

**Table S2. Metabolic parameters and outcomes for control subjects and diabetic patients.**

| Characteristics | Control | Diabetes | p |
| --- | --- | --- | --- |
| N | 769 | 769 | - |
| Male (%) | 266 (34.6) | 266 (34.6) | - |
| BMI | 23.38$\pm$3.09 | 24.22$\pm$3.48 | <0.001 |
| ANGPTL8 (pg/mL) | 581.20$\pm$299.54 | 618.82$\pm$381.08 | 0.03 |
| HbA1c (%) | 5.56$\pm$0.34 | 7.17$\pm$1.97 | <0.001 |
| FPG (mmol/L) | 5.05$\pm$0.47 | 7.92$\pm$2.74 | <0.001 |
| 2h PG (mmol/L) | 5.72$\pm$1.10 | 12.17$\pm$4.84 | <0.001 |
| Fasting insulin (pmol/mL) | 6.09$\pm$ 5.21 | 10.13$\pm$11.10 | <0.001 |
| HOMA-IR | 1.39$\pm$1.27 | 3.65$\pm$4.30 | <0.001 |
| HOMA-$\beta$(%) | 76.94$\pm$142.63 | 56.08$\pm$79.83 | <0.001 |
| Fasting HDL (mmol/L) | 1.40$\pm$0.35 | 1.43$\pm$0.38 | 0.07 |
| Fasting LDL (mmol/L) | 2.62$\pm$0.74 | 3.00$\pm$0.86 | <0.001 |
| Fasting TG (mmol/L) | 1.58$\pm$1.45 | 1.87$\pm$1.49 | <0.001 |
| Cholesterol (mmol/L) | 4.75$\pm$0.95 | 5.22$\pm$1.06 | <0.001 |
| eGFR (mL/min/1.73 m^2^) | 115.46$\pm$21.97 | 108.35$\pm$20.72 | <0.001 |
| Creatinine (μmol/L) | 63$\pm12$ | 66$\pm$21 | <0.001 |
| Hypertension | 478 (62.2) | 583 (75.8) | <0.001 |
| Hyperlipidaemia | 287 (37.3) | 435 (56.6) | <0.001 |
| Outcomes | | | |
| Primary outcome | | | |
| Death | 19 (2.5) | 56 (7.3) | <0.001 |
| Secondary outcomes | 44 (5.7) | 91 (11.8) | <0.001 |
| MACE | 34 (4.4) | 62 (8.1) | 0.004 |
| HF | 3 (0.4) | 12 (1.6) | 0.03 |
| Renal dysfunction | 7 (0.9) | 23 (3.0) | 0.005 |

Abbreviation: BMI, body-mass index; HbA1c, glycated haemoglobin A1c; FPG, fasting plasma glucose; 2h PG, 2 h plasma glucose concentration; HOMA-IR, homeostasis model assessment of insulin resistance; HOMA-β , homeostasis model assessment of β cell function; HDL, high density lipoprotein; LDL, low density lipoprotein; TG, triglycerides; ALT, alanine transaminase; AST aspartate aminotransferase; eGFR, glomerular filtration rate; MACE, new-onset major adverse cardiovascular events; HF, hospitalization for heart failure.

**Table S3. Partial correlations between ANGPTL8 levels and clinical variables in control subjects and diabetic patients.**

|  | Control | | | | | |  | Diabetes | | | | | |
| --- | --- | --- | --- | --- | --- | --- | --- | --- | --- | --- | --- | --- | --- |
|  | Model 1 | | Model 2 | | Model 3 | |  | Model 1 | | Model 2 | | Model 3 | |
|  | r | p value | Partial r | p value | Partial r | p value |  | r | p value | Partial r | p value | Partial r | p value |
| Age | 0.423 | 0.000 | 0.418 | 0.000 | 0.421 | 0.000 |  | 0.204 | 0.000 | 0.165 | 0.000 | 0.177 | 0.000 |
| BMI | 0.061 | 0.090 | 0.131 | 0.000 | 0.101 | 0.005 |  | -0.112 | 0.002 | -0.044 | 0.219 | -0.074 | 0.041 |
| WHR | 0.055 | 0.127 | 0.017 | 0.646 | 0.013 | 0.730 |  | -0.062 | 0.086 | -0.056 | 0.119 | -0.065 | 0.072 |
| SBP | 0.168 | 0.000 | 0.029 | 0.429 | 0.015 | 0.676 |  | 0.031 | 0.389 | -0.022 | 0.536 | -0.014 | 0.693 |
| Duration of diabetes | - | - | - | - | - | - |  | 0.063 | 0.081 | 0.083 | 0.021 | 0.078 | 0.031 |
| HbA1c | 0.095 | 0.008 | -0.009 | 0.794 | -0.021 | 0.568 |  | -0.032 | 0.379 | 0.035 | 0.332 | 0.029 | 0.418 |
| FPG | 0.006 | 0.860 | -0.018 | 0.625 | -0.026 | 0.477 |  | 0.013 | 0.709 | 0.067 | 0.064 | 0.0762 | 0.089 |
| 2h PG | 0.046 | 0.204 | 0.011 | 0.770 | 0.003 | 0.943 |  | 0.051 | 0.161 | 0.081 | 0.024 | 0.078 | 0.032 |
| Fasting insulin | -0.002 | 0.951 | 0.038 | 0.294 | 0.021 | 0.559 |  | -0.018 | 0.625 | 0.020 | 0.583 | -0.002 | 0.967 |
| HOMA-IR | -0.004 | 0.922 | 0.032 | 0.379 | 0.015 | 0.688 |  | -0.003 | 0.926 | 0.038 | 0.298 | 0.013 | 0.716 |
| HOMA-$\beta$ | 0.004 | 0.907 | 0.001 | 0.988 | -0.006 | 0.877 |  | -0.064 | 0.077 | -0.038 | 0.288 | -0.047 | 0.195 |
| HDL | 0.008 | 0.817 | -0.039 | 0.278 | 0.005 | 0.900 |  | -0.091 | 0.012 | -0.150 | 0.000 | -0.086 | 0.017 |
| LDL | 0.055 | 0.126 | 0.028 | 0.442 | 0.035 | 0.330 |  | -0.090 | 0.013 | -0.070 | 0.051 | -0.036 | 0.319 |
| TG | 0.058 | 0.110 | 0.091 | 0.012 | 0.069 | 0.057 |  | 0.068 | 0.059 | 0.123 | 0.001 | 0.030 | 0.407 |
| Cholesterol | 0.074 | 0.039 | 0.038 | 0.293 | -0.024 | 0.501 |  | -0.065 | 0.072 | -0.044 | 0.219 | 0.019 | 0.598 |
| ALT | 0.007 | 0.849 | 0.041 | 0.258 | 0.030 | 0.405 |  | 0.066 | 0.068 | 0.079 | 0.029 | 0.072 | 0.046 |
| AST | 0.056 | 0.124 | 0.016 | 0.663 | 0.016 | 0.662 |  | 0.150 | 0.000 | 0.126 | 0.000 | 0.134 | 0.000 |
| eGFR | -0.225 | 0.000 | -0.135 | 0.000 | -0.123 | 0.000 |  | -0.126 | 0.000 | -0.137 | 0.000 | -0.130 | 0.000 |
| Creatinine | 0.215 | 0.000 | 0.118 | 0.001 | 0.108 | 0.003 |  | 0.154 | 0.000 | 0.107 | 0.003 | 0.096 | 0.008 |

* when a variable was calculated in the partial correlation, it would not be included in adjustment model.

Model 1 was unadjusted.

Model 2 was adjusted by age, sex and BMI.

Model 3 was adjusted by all variables in model 2 plus HDL, LDL, TG, cholesterol.

Abbreviation: BMI, body-mass index; WHR, waist hip rate; SBP, systolic blood pressure; HbA1c, glycated haemoglobin A1c; FPG, fasting plasma glucose; 2h PG, 2 h plasma glucose concentration; HOMA-IR, homeostasis model assessment of insulin resistance; HOMA-β, homeostasis model assessment of β cell function; HDL, high density lipoprotein; LDL, low density lipoprotein; TG, triglycerides; ALT, alanine transaminase; AST aspartate aminotransferase; eGFR, glomerular filtration rate.

**Table S4. Predictive** **values** **for all-cause mortality in combination with ANGPTL8 in QFrailty score.**

| Variables | AUC | SE | p for individual | 95% CI | | p for comparison |
| --- | --- | --- | --- | --- | --- | --- |
|  |  |  |  | Lower bound | Upper bound |  |
| All participants | | | | | |  |
| QFrailty score | 0.59 | 0.038 | 0.013 | 0.51 | 0.66 | 0.000 |
| QFrailty score + ANGPTL8 | 0.71 | 0.031 | 0.000 | 0.65 | 0.77 |  |
| Diabetic patients | | | | | |  |
| QFrailty score | 0.59 | 0.044 | 0.032 | 0.50 | 0.67 | 0.000 |
| QFrailty score + ANGPTL8 | 0.70 | 0.037 | 0.000 | 0.63 | 0.78 |  |
| Control subjects | | | | | |  |
| QFrailty score | 0.57 | 0.073 | 0.304 | 0.43 | 0.71 | 0.01 |
| QFrailty score + ANGPTL8 | 0.71 | 0.058 | 0.002 | 0.59 | 0.82 |  |

Abbreviation: AUC, area under the curve; CI, confidential interval; SE, standard error

**Figure S1. Flow diagram for the study population selection.**


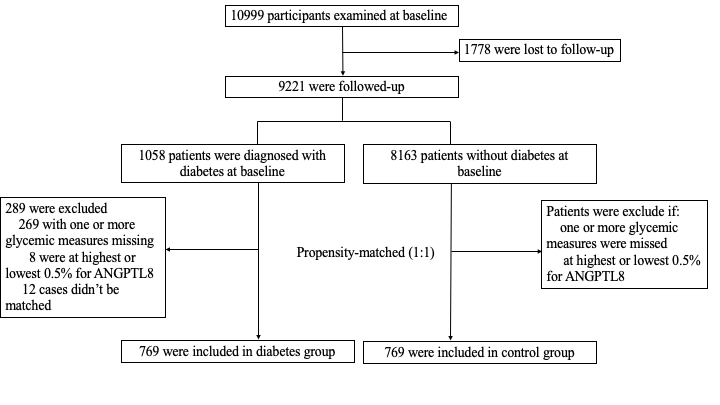

Supplement: Supplementary file 1 — Additional file 1: Appendix 1. Predictor variables for QFrailty score. Table S1. Characteristics of patients with diabetes. Table S2. Metabolic parameters and outcomes for control subjects and diabetic patients. Table S3. Partial correlations between ANGPTL8 levels and clinical variables in control subjects and diabetic patients. Table S4. Predictive values for all-cause mortality in combination with ANGPTL8 in QFrailty score. Figure S1. Flow diagram for the study population selection. [file 12933_2020_1103_MOESM1_ESM.docx]
